# VOCATIONAL ASSESSMENT AND INDIVIDUAL DEVELOPMENT PLANNING IN DIVERSITY-FOCUSED UNDERGRADUATE MENTORSHIP

**DOI:** 10.1093/geroni/igac059.3074

**Published:** 2022-12-20

**Authors:** Sheri Thompson

**Affiliations:** UC San Diego, La Jolla, California, United States

## Abstract

The UC San Diego MADURA Mentorship Program, funded the National Institute on Aging, strives to improve diversity in Aging/Alzheimer’s Disease and Related Dementias (ADRD) research and clinical practice. Goals include improved academic success of undergraduate URM trainees, and facilitating graduate/medical school applications and entry into Aging/ADRD research or clinical careers. Mentees receive paid research experience, training, Program and lab-based faculty supervision, peer support, seminars, and professional development opportunities. To enhance educational and career planning, MADURA incorporated Individual Development Plan (IDP) use, in 2022. IDP training by a Career Services presenter was followed by interactive IDP completion during small group break-outs, with ongoing revisions occurring throughout the quarter. Further, all trainees were offered formal vocational assessment (Strong Interest Inventory®, College Edition). 21 trainees participated and some engaged in individual 30-minute feedback sessions. All mentors were asked to incorporate IDP and vocational assessment discussions into year-end professional development meetings with mentees. Data to be presented includes: Student evaluation of IDP training; prior vocational assessment access; student and mentor ratings of IDP and vocational assessment feasibility, utility and satisfaction; and cohort vocational profile characteristics. Discussion includes implementation challenges, benefits and limitations of IDP and vocational assessment use, and future research suggestions.

